# Medical Items and News of Interest to the Physician

**Published:** 1873-10

**Authors:** 


### For the Use of Physicians.

CULLED FROM THE STANDARD MEDICAL JOURNALS OF THE WORLD.

NOTE.—These formulæ are intended only for the regular practitioner of medicine, and should be employed only by him, or under his immediate supervision.

**Bright's Disease.**

The general principles of treatment embrace the administration of tonics and diuretics. The best tonics are quinine and tr. chloride of iron; the best diuretic is thought to be the infusion of digitalis.—*Med. Record.*

**Diabetes.**

M. Devergie (*LaFrance Medicale*) recommends the employment of an arsenical treatment in the case of diabetes, and has satisfied himself of its good effects even when no restrictions were laid upon the diet and farinaceous articles were freely taken.

**Anti-Neuralgic Snuff.**

Dr. F. Scriffignano recommends in facial neuralgia, especially of the periodic form, the use of snuff containing fifty centigrammes of quinine to one gramme of tobacco. Pinches of this mixture are to be taken at frequent intervals during three consecutive days.—*Boston Med. and Surg. Journal.*

**Subacute Pleurisy.**

Patients who present their credentials and are booked "effusion into the pleural cavity—large quantity," are tapped at once, and placed upon tonics, quinine and iron. Diuretics are not used with any great degree of confidence. When the effusion is moderate, tonics alone are used.—*Ibid.*

**Erysipelas.**

For a local application the liq. plumbi et opii. is used, and for internal administration quinine and iron, and the quinine is thought to be especially serviceable. It may have been noticed that the administration of quinine and iron in some of the hospitals is not so steadily adhered to as has been wont to be the case with the profession in general. Of their value in this disease, it will be difficult to convince the profession, notwithstanding some of the cases which are treated without the administration of these remedies seem to do equally as well as those who receive it.—*Ibid.*

**For Burns.**

At the Charity Hospital, New York, according to the *Medical Record*, a favorite lotion for burns is made by this formula:—

Sulphate zinc, grs. xv.

Co. spts. lavender, ʒ i.

Aqua, one pint, M.

**Dressing for Ulcers.**

At the same institution a dressing which is said to serve a most admirable purpose for any ulcerated surface which may need a soothing and slightly stimulating application, is the ceratum resinæ et bals. Peruvian. It is usually employed in the proportion of one part of balsam to four of ceratum.

**Perineorrhaphy.**

Dr. E. S. Bunker, of Brooklyn, N. Y., in *Medical Record*, recommends the use of horse hair for sutures in the operation named. He says it is much easier of management than silver wire, causes no suppuration along its course, and does not chafe or excoriate the parts, nor catch in the patient's linen.

**Chapping of the Skin.**

℞

Oxide of zinc, gr. xx.

Tannic acid, gr. xv;

Glycerine, ʒ iv;

Tinct. of benzoin, ʒ ss;

Camphor, gr. xv. M.

Apply locally.—*Boston Med. and Surg. Journal.*

**Opium Antidotes.**

Dr. H. G. Wallace, in the *Med. and Surg. Reporter*, gives the following formula as a valuable antidote for the "opium habit:"

℞

Potassium bromide, gr. viij.
Fougera's cod-liver oil, ℥ ss.

S. Take the above, in one dose, three times daily.

**Night Sweats.**

The most ancient and venerable remedy for night-sweats is aromatic sulphuric acid, in infusion of cinchona, or serpentaria. According to the *Pacific Med. & Surg. Jour.*, the best remedy is the following:

℞

Oxidi zinci, gr. xxx;
Ext. hyoscyami, gr. xv,

M. fiat pillula No. x. Sig: Take one at bedtime.

**Action of the Bromides in Pruriginous Affections.**

Dr. Gueneau (de Mussy) extols the efficacy of the bromides applied locally in pruriginous affections of the external and internal integuments. He recommends their use, especially in pruritus vulvæ, and employs the bromides either in ointment or solution.

**Chronic Eczema.**

Dr. De Montyma recommends the use of tincture of iodine in the treatment of the chronic eczema, and intertrigo of the genitals, more especially combined with the use of one part of corrosive sublimate to 250 of water, a few drops of spirit being used to dissolve the corrosive sublimate.—*Med. & Surg. Reporter.*

**Chronic Diarrhœa.**

Rayer, (*Union Medicale,*) advocates the combination of cinchona, charcoal, and bismuth in the management of chronic diarrhœa, in these proportions :

℞

Subnitrate of bismuth, ʒ j;

Cinchona, yellow, powdered, ʒ ss;

Charcoal, vegetable, ʒ j:

M. Chart. No. xx. Sig. One powder, two or three times daily, during the intervals between meals.

**Bleached Tincture of Iodine.**

Sulphite of soda will discolor iodine without diminishing, but rather increasing its effect. The *Medical Press and Circular* gives a formula for the combination, viz. : Tinct. iodine, glycerine, pure, āā ℥ i ; sodæ sulphitis, ℥ i. M. Rub the sulphite to a powder, in a small mortar, and add the glycerine gradually ; then pour in the tincture and triturate gently, until a solution is effected and the solution assumes an amber color.—*Canada Lancet.*

**Constipation.**

M. Isnard recommends arsenic in the treatment of constipation, especially in the cases of females. Although any preparation of arsenic seems to answer, M. Isnard prefers, ordinarily, arsenious acid. Three grains being dissolved in two and one-half pints of water, a coffee spoonful will represent one milligramme of acid. A medium dose is six to ten coffee spoonfuls daily, in three doses, preferably taken at meal-time, in water or wine, and increased gradually in quantity.—*Le France Med.*

**Inhalation in Gonorrhœa.**

In a recent article on the "inhalations of balsams," Prof. Dittel, of Vienna, reported marked success for his method in two cases of gonorrhœa, and cured one in twenty-five and the other in eighteen days. He believes with Wickart that the anti-blenorrhagic action of these balsams is not due to the ethereal oils but rather to the resinions principles. These latter are always detected in the urine during the treatment, while there is no trace of the former.—*Med. & Surg. Reporter.*

**Belladonna in Typhoid Fever.**

Dr. Pilcher, Passed Asst. Surgeon in the Navy, says that in from twenty-four to forty-eight hours after the first administration of belladonna in typhoid fever, delirium, coma, and subsultus vanish, and are succeeded by calmness and clearness of the intellect, by natural sleep, and complete control of all the voluntary muscles; diarrhœa is checked, and healthy and consistent evacuations are established.

**To Remove Adhesive Plaster.**

Every surgeon, doubtless, is familiar with the appearance of a part which has been enveloped in adhesive plaster, after the straps have been removed. The appearance is not one in very good keeping with cleanly and neat surgical dressing. The portion of the plaster which is adhering to the skin may be quickly and completely removed by the use of oil of turpentine and sweet oil. Use a little more than half turpentine. This compound, carefully rubbed over the parts with a bit of cloth or sponge, and then washed off with warm soap-suds, will leave the surface as clean as nature ever intended.

**Podophyllin in Constipation.**

Dr. Constantin Paul lately read a paper on this drug at the Société de Thérapeutique, which has been published in the *Gazette Medicale de Paris*. He considers this remedy one of the most reliable in habitual constipation. He began by combining it with belladonna as advised by Trousseau and others. When belladonna did not agree he substituted hyoscyamus. He has now discarded all adjuvants, and recommends a small dose of podophyllin made into a pill with honey, to be taken every night. It usually produces a single evacuation each morning. Should there be more effect after a few days, he omits the dose for a night or two.

**Croup.**

Dr. W. W. Parker, of Richmond, Va., (*Virginia Clinical Record,*) relates a case of croup in which inhalations of lime proved efficacious. The most dense vapor is not at all unpleasant, and can be borne as well as the ordinary atmosphere of a heated room.

This is the plan of treatment we have been accustomed to for years, and have always found it reliable as a means of dissolving the membrane and speedily relieving all unpleasant indications.—*Ed. Bistoury.*

**Coitre in Georgia.**

According to the *Georgia Medical Companion,* there is a neighborhood on the head waters of Lott's Creek, Scarboro', Georgia, containing about fifty families, and spread over a territory of about five to seven miles, the female portion of which seems to be predisposed to this disease. There is rarely a female that has arrived at puberty that is not afflicted with it. It has been so with almost all the women raised there. There never was a male known to have it in that settlement. The disease yields readily to the iodine treatment. The soil is sandy; growth principally pine, interspersed with broad leaf jack. The drinking-water is obtained from wells, the average depth of which is about twenty feet. The locality is noted for its extreme healthfulness in other respects.

**Diagnosis of Lipoma.**

Monsieur Montmeja publishes a new observation of symmetrical lipomas collected in the service of M. Panas, and calls the attention of surgeons to a peculiar characteristic of lipomas which admits, in many cases, the establishment of their presence when the diagnosis is attended with difficulties. This characteristic is the property possessed by fatty tumors of becoming harder by the action of cold. In the cadavar fatty tumors are very hard; a lipoma removed from a living body hardens in proportion as it cools. It is possible to cool by ice or atomized either, a tumor of doubtful nature, and if to the touch there is manifest induration following the action of the cold, there is every reason to predicate the existence of lipoma. Medical treatment is powerless to effect the removal of lipoma; but some of small size have been known to disappear under the influence of continuous electrical currents, and M. Montmeja cites one case favoring this mode of treatment.—*Rev. Medico photo. des Hop.*

**Indolent Ulcers.**

At a discussion before the Rock River Medical Society, of Wisconsin, the following treatment was recommended:

℞
Potass. iodidi,
Potass. bromidi, āā ʒv;
Syr. ferri iodidi, ℥ ij;
Aquæ, ℥ vj. M.

Sig: One teaspoonful three times a day, after meals.

In local treatment perfect rest is recommended. If inflammation is not yet subdued, apply fomentation of chamomile infusion. If edges of ulcer are hard and indurated, strapping will be useful in promoting the absorption of the plastic material. The general dressing should consist of carbolized linseed oil, prepared in the proportion of one part of carbolic acid to twelve of linseed oil.—*Med. and Surg. Reporter.*

**Cholera--by A. C. Craig, M. D., Ghent, Ky.**

Asiatic cholera has again visited the United States, and has made its appearance about two months earlier than it did in 1866. During the last epidemic of this fatal malady, I was the Resident Physician of the Cincinnati Hospital, and my opportunities for observing the disease in all its stages, and testing the efficacy of various remedies in its treatment, were by no means limited. In almost every case of cholera there is a premonitory diarrhœa lasting from a few hours to several days, which, if properly treated, is readily cured. In time of cholera every movement of the bowels, after what is customary and natural, must be regarded as a tendency toward the disease, and after diarrhœa is established, the patient should go to bed and have a physician summoned immediately. The following remedy has been used by the writer in hundreds of cases of cholera, and it will arrest almost every case of the disease in the *first stage.*

Take of laudanum, spirit of camphor and tincture of ginger, each four fluid drachms; tincture of capsicum and chloroform, each two fluids drachms; pure brandy, two fluid ounces. Mix. Dose for an adult, a teaspoonful in ice water after each movement of the bowels.

The above remedy will prove as effectual in the rice-water stage as in the first or prodromic stage.

**Biliary Calculus.**

Schiff admits that these calculi are formed by cholesterin, not because this substance is formed in too great abundance, but because the bile does not contain the principles which maintain it in solution. These are the cholates and choleates of soda and potassa, more than the alkalinity of the bile which dissolves the cholesterin. Schiff, therefore, advises the administration of eight grains choleate of soda to be given twice daily, and increased until "saturation" is indicated by irregularity of the pulse, which becomes slow during repose and accelerated by the least effort. The dose may then be diminished, but not entirely suspended, a considerable time, a week at least, being required for the remedy to produce amelioratian of the symptoms.—*Imparziale & Gaz. Heb.*

---

**Malignant Pustule.**

Dr. Belles, practicing in the districts of Bija, Portugal, where malignant pustule is very frequent, in consequence of the number of cattle raised there, says he has treated hundreds of cases of this affection without having noted a single death, no matter how advanced the case was at the time of operating. The treatment employed by Dr. Belles consists in crucial incision of the boil throughout its whole extent, in depth as well as breadth, and cauterization with chloride of antimony, repeated until bleeding ceases. The gangrenous tissue is completely insensible to both cutting and cauterization. When the operative measures have been postponed until extensive disorganization of the tissues has occurred, the separation of the eschar is retarded by local atony, which the author counteracts by means of poultices of meal and tapioca cooked in wine. —*Gaz. Med. de Paris.*

---

**Facial Erysipelas.**

Prof. D. M. Salazar, of the hospital National, Madrid, reports that he has cured eight cases of facial erysipelas in 48 hours by glycerine of borax. Notwithstanding the rapidity with which the affection disappeared, there were no consecutive pathological affections. In one case the disease had existed three days before treatment was commenced, and there was bilious vomiting, intense cephalgia, high fever, inflammation of the entire face, and some phlyctenulæ in the vicinity of the right lower eye-lid and the root of the nose. He applied the solution to the deceased parts with a brush and then covered them with a mask of raw cotton. After 24 hours all the symptoms, local and general, were notably diminished, and the next day all the phlyctenulæ had disappeared and desquamation was commencing. All medication was then discontinued, except that a decoction of sambucus and althæa was used as a wash to favor the desquamation.—*El Amfit Anat. Espan., From Med. Record.*.

---

**Insanity.**

Dr. D. H. Kitchen, (*Am. Jour. Insanity*) in an excellent article upon this subject, speaks of the valuable experiments with *conia* hypodermically administered by the attending physician of West Riding Lunatic Asylum. Twelve cases are related in which the drug was successfully given. His conclusion on its action is as follows:—

1st. Muscular relaxation. 2d. Duration in proportion to dose. 3d. Physiological effect in proportion to purity of the article used. 4th. The brain is not affected by conium. 5th. Pulse and temperament both reduced after a full dose. 6th. A gentle perspiration covers the whole body as soon as the physiological effects are observed. 7th. No appreciable effect upon any of the secretions. 8th. Quietness lasts from two to four hours, and then disappears, leaving only a sense of lessened muscular energy. 9th. Conium, not acting on the brain, may safely be given in all febrile diseases. 10th. Conium, when applied to the skin, causes slight redness.

---

**The Treatment of Fever by Carbolic Acid.**

Assistant-Surgeon McNarrey (*Mardas Medical Journal*, Jan. 1, 1873) warmly recommends the use of carbolic acid to replace quinine in the treatment of intermittent fevers. He gives a tabular view of his cases which he summarizes thus:—

"I administered it in seventy-six cases of fever of different types, with most marked success, and in no single instance with bad results, but, owing to the great amount of work consequent upon the epidemic, it was impossible to enter every case that occurred fully in the journal, although brief statements were made of each and the result corresponded with those now recorded. I gave it in doses ranging from five to twenty minims, and in those cases where I observed a tendency to relapse I at once increased the dose. It was administered in bitter infusions of either gentian, calumba, or chiretta, three times a day.

"The Carbolic acid I employed was Calvert's No. 1 for internal use.

"Having now had ample opportunity of judging of the effects of this remedy, and finding its success to have exceeded my expectations, I trust that the information I have given will be sufficient to induce others to adopt it in similar cases.

"I have little doubt that the ease with which it can be procured, and the small amount of trouble entailed in its administration, added to its trifling cost, and the immense saving to government that would ensue from its being largely substituted for quinine, will recommend it as a valuable febrile remedy; and I do not think a careful trial of it will fail to prove satisfactory."

---

**Turpentine in Erysipelas.**

Dr. Leonardi reports (*Gaz. Med. de Paris*, from *Journ. de Med.*, vol. lv. p. 316) a case of a woman, aged forty-two, whose health was generally good, and who slept in the open air. On awaking she experienced severe pain in her head and neck. On the following day she had a shivering fit, followed by fever, and erysipelas appeared in the neck. On the second day the chest was attacked as well as the ears, which attained an immense size.—M. Leonardi, on seeing the patient, prescribed the application of oil of turpentine twice a day to all the parts affected, and some aperient medicine. At the end of three days the affected parts had recovered their normal size, and the desquamation of the epidermis was the only trace of an attack of erysipelas so severe that it was at one time thought it would prove fatal.

He also gives another case of a scrofulous child, eight years of age, who, having been long exposed to the sun, began to suffer from pain over the whole right side of the chest. The next day the whole face was affected, and the nose and ears, greatly swollen, were covered with phlyctenulæ. The febrile symptoms were intense, the tongue dry, and all the symptoms of severe erysipelas of the face were present. Here, again, M. Leonardi prescribed embrocations of essential oil of turpentine twice a day, and internally some enemata with oil of turpentine. Several large lumbricoid worms were expelled, and on the forth day the cure was complete.

The efficacy of applications of oil of turpentine, the author goes on to say, has been frequently verified in cases of traumatic erysipelas. The above cases show that it is equally serviceable in cases of spontaneous or idopathic erysipelas.

---

**Intermittent Fever.**

Dr. J. Lewis Smith, of Bellevue Hospital, Medical College, N. Y., (*Medical Record*,) is in the habit of prescribing for fever and ague in children as follows :

To an infant of two years I prescribe one grain of sulphate of quinine or the equivalent of sulphate of cinchona, three times daily, till all symptoms of the ague have disappeared ; then twice a day during the subsequent week, and afterwards once a day for some days, and finally twice or thrice a week. It is only by the protracted use of the drug in occasional doses that the return of the intermittent can be prevented. It is important in administering these sulphates to infants to employ a vehicle which will, as far as possible, disguise the bitterness. I have found nothing so good for this purpose, as the syrup of one of the berries, as in the following formula :—

℞

Cinchoniæ sulphat., gr. xvj ;
Acid sulphur, dilut., gtt. xxiv;
Syr. rubi idæi, (raspberry,) ℥ij. M.

Sig. One teaspoonful contains one grain of cinchoniæ.

If the patient, when presented for treatment is pallid from the long continuance of the ague, or from other cause, physicians are in the habit, very properly, of prescribing iron in addition to the specific.

---

**Syphilitic Skin Diseases,**

Dr. McCall Anderson, (*Med. News & Library*), lays down the following rules with regard to the employment of iodide of potassium in the treatment of syphilitic skin diseases:—

1st. The longer the interval which has elapsed between the contraction of the syphilitic taint and the development of the eruption, the more confidently may we substitute it for mercury.

2d. If the patient is cachectic, it is, as a rule, to be preferred to mercury, except in recent cases of syphilis, when the mercurial vapor bath, or some such treatment, is more likely to prove successful.

3d. The more extensive the tertiary eruption, the more certain it is to yield to the iodide of potassium; although to this rule there are numerous exceptions.

4th. If there is any tendency to syphilitic diseases of the nostrils or neighboring parts, iodide of potassium should be withheld, or given with great caution, for, if it produces coryza, it is very apt to aggravate the morbid condition of the parts.

5th. It should be given in full doses. It is generally advisable to prescribe it in combination with a bitter, and, in cachectic patients, a little iron is a valuable addition, as in the subjoined prescription:

℞
Ammonia—citrate of iron, ʒ iij;
Iodide of potassium, ℥ j;
Syrup of ginger, ℥ vj;
Comp. infus. of gentian, ℥ viij;
Water to ℥ xxiv. M.

S. A tablespoonful in a large wine-glass of water thrice daily.

---

**St. Luke's Hospital, N. Y.**

The following formulæ and plans of treatment are adopted at the hospital mentioned, and are published in the *Medical Record:*

Sprains.—These cases are immediately, as a rule, treated to a plaster of paris splint, with the precaution taken to pad the limb well with cotton before making the application.

Delirium Tremens.—Most of these patients, it is said, will require some stimulants. A man was receiving ℥ j. three times a day. Hydrate of chloral, bromide of ammonium and potassium, and other remedies which naturally suggest themselves to the mind of the practitioner, are used if the patient requires anything besides the care and quiet of the hospital to secure sleep.

Pneumonia.—The plan of treatment ordinarily adopted in this disease is to give the patient three grains of quinine three times per day, simply for its supporting effect; if the temperature gets up to 104° or 105°, reduce it by rubbing them over with cacao butter, which operates very nicely [indeed when used for that purpose, feed them well, and that is about all. The treatment is simple but the results are gratifying.

Scarlatina.—The remedial measures employed in this affection are in keeping with the generally received doctrine that it belongs to a class of diseases called self-limited; hence all the duty of the physician consists in guiding his patients among the shoals and rocks to which they are exposed during their perilous voyage. About ʒi. of cacao butter is used as an unction twice a day, to relieve the high temperature, and after this the symptoms are met as they arise. This article of butter of cacao receives a hearty recommendation as an agent to be used for the reduction of the temperature in this class of cases.

Burns.—Gunpowder burns are brushed with bichloride of mercury, one grain to the ounce of water, with the addition of one drachm of tr. benzoin. This is an old prescription here and is supposed to be especially serviceable in connection with gunpowder burns. Oxide of zinc ointment has the practical recommendation of an old member of the fire department.

Night Sweats in Phthisic.—A remedy commonly employed for the relief of this symptom is: Fluid Extract Ergot in drachm doses at night. In some cases the patients vomit the remedy, but it is said to work exceedingly well in a large majority of cases. Hydrate of chloral, given in grs. xx doses about two hours before the time for the sweating to commence, is another plan. Another method is to awaken the patient a little before the hour at which the sweating commences, have him wash himself and take a little lunch.

Surgical Dressing.—In all those cases where it is desirable to keep up support and pressure, and at the same time permit the free escape of all discharges from the wound, or ulcer, or whatever it may be, the ordinary mosquito netting used for a bandage meets all the indications. Bundling dressings are avoided in this way, the parts are kept cool, the discharge goes on unrestrained, and at the same time support is maintained. If the discharge is considerable, a pad of oakum may be placed beneath the parts to secure the discharge, thus ensuring perfect cleanliness. This netting serves an admirable purpose in dressing large abscesses; for instance, when compression and free discharge are to be associated.

Acute Articular Rheumatism.—When one of these patients are brought in with joints painful, swollen, and tender, and motion about suspended, in short, with all the phenomena of an attack of acute articular rheumatism, he is immediately placed upon a treatment which consists in the administration of iodide of potash in fifteen grain doses every two hours, and sulphate of quinia gr. x, alternately, every two hours.

This is continued until the acute symptoms subside. It is expected that this will take place within fifty-six hours, and is discontinued at the end of this time in case the acute symptoms do not yield. In most cases the acute symptoms are completely subdued within twenty-four or forty-eight hours, and the patients feel comparatively comfortable. This, with a certain sense of propriety, might be regarded as heroic treatment, yet the results sanction and commend its adoption. The local treatment scarcely goes beyond covering the joints with cotton. Later in the treatment colchicum enters, and is regarded as a useful adjuvant to the salts which are ordinarily employed. It is said that the salts do much better when combined with colchicum, than when they are used alone.

Testing Bread and Flour for Alum.—Prof. L. A. Bunchner says that bread and flour free from alum gives, when touched with an alcoholic solution of extract of campeachy wood, a brownish yellow spot, but if not less than 1 or 2 per cent. of alum is present, a grayish violet color is produced; ½ per cent. alum gives a reddish yellow with a grayish blue border; and blue spots are distinctly visible with a magnifying glass; with only ¼ per cent. of alum, the grayish blue edge could not be discerned, but a careful examination with a glass discovered tiny blue dots in the yellow spot.

Opium Eating.—The Legislature of Kentucky, in order to check the practice of opium eating, which is greatly on the increase, has just passed a bill that on the affidavit of two respectable citizens, any person who, through the excessive use of opium, arsenic, hasheesh, or any drug, has become incompetent to manage himself or his estate, may be confined in any asylum and placed under guardianship, as in the case of habitual drunkards or lunatics.—*Med. & Surg. Rep.*

